# Gaussians for Electronic and Rovibrational Quantum Dynamics

**DOI:** 10.1021/acs.jpca.4c00364

**Published:** 2024-04-30

**Authors:** Aleksander P. Woźniak, Ludwik Adamowicz, Thomas Bondo Pedersen, Simen Kvaal

**Affiliations:** † Faculty of Chemistry, University of Warsaw, Pasteura 1, 02-093 Warsaw, Poland; ‡ Department of Chemistry and Biochemistry, University of Arizona, 1306 E University Blvd, Tucson, Arizona 85721-0041, United States; ¶ Hylleraas Centre for Quantum Molecular Sciences, Department of Chemistry, University of Oslo, P.O. Box 1033 Blindern, N-0315 Oslo, Norway

## Abstract

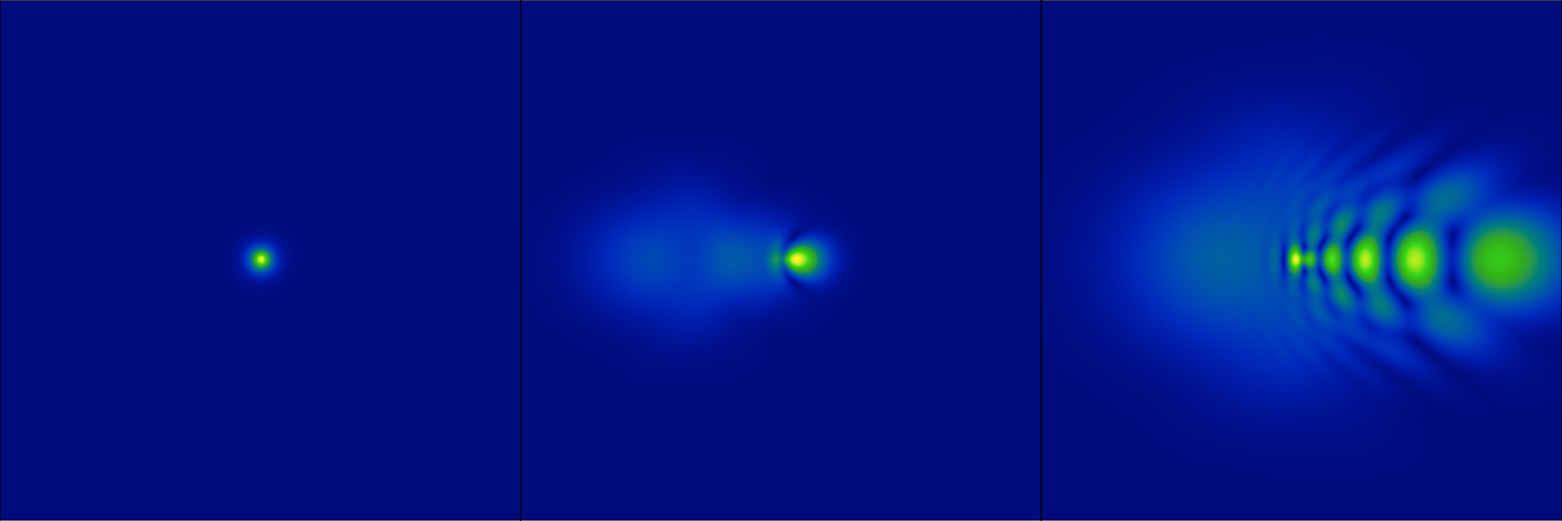

The assumptions underpinning the adiabatic Born–Oppenheimer (BO) approximation are broken for molecules interacting with attosecond laser pulses, which generate complicated coupled electronic-nuclear wave packets that generally will have components of electronic and dissociation continua as well as bound-state contributions. The conceptually most straightforward way to overcome this challenge is to treat the electronic and nuclear degrees of freedom on equal quantum-mechanical footing by *not* invoking the BO approximation at all. Explicitly correlated Gaussian (ECG) basis functions have proved successful for non-BO calculations of stationary molecular states and energies, reproducing rovibrational absorption spectra with very high accuracy. In this Article, we present a proof-of-principle study of the ability of fully flexible ECGs (FFECGs) to capture the intricate electronic and rovibrational dynamics generated by short, high-intensity laser pulses. By fitting linear combinations of FFECGs to accurate wave function histories obtained on a large real-space grid for a regularized 2D model of the hydrogen atom and for the 2D Morse potential, we demonstrate that FFECGs provide a very compact description of laser-driven electronic and rovibrational dynamics.

## Introduction

With the advent of new technology for manipulating atoms and molecules with intense ultrashort (attosecond and femtosecond) laser pulses, there is an urgent need for further development of accurate and reliable quantum-dynamics (QD) tools for simulations of events involved in such manipulations. Such simulations are needed both to guide the design of experiments and to ensure the correct interpretation of observations. Atomic and molecular QD simulations involving interaction of these systems with ultrashort intense laser pulses can be carried out by integrating the time-dependent Schrödinger equation (TDSE) on a real-space grid or by using an expansion of the wave function of the system in terms of basis functions whose linear and nonlinear parameters are adjusted along with the number of basis functions during the propagation. The focus of this work is an analysis of the necessary features such basis functions must possess to be effective for simulating laser-induced dynamics of an atomic or molecular system.

The dynamics induced by the interaction of intense electromagnetic radiation with nuclei and electrons of a molecule necessitate an accurate account of the coupling of these two types of particles. The broad intensity distribution in the frequency domain of ultrashort laser pulses implies that a large number of bound and continuum states are involved in the dynamics, including rovibrational, electronic, and collective states, where the motions of both nuclei and electrons are simultaneously excited. To describe such an intricate situation, the separation of the nuclear and electronic motions—a hallmark of the adiabatic Born–Oppenheimer (BO) approximation^1,2^—must not be assumed and, ideally, all particles forming the system should be treated on an equal footing. Such an approach is tested in this work using two simple 2D models. Also, an extension of the approach to simulate the laser-induced dynamics of attosecond atomic and molecular events involved in attosecond experiments^3−5^ is discussed.

The coupled nuclear-electronic motion is highly correlated, as the electrons, particularly the core electrons, generally follow the nuclei very closely, and the nuclei stay apart from each other due to their strong Coulomb repulsion and large masses. The situation is markedly different for the electrons whose wave functions, due to much lower masses, more significantly overlap. To best describe these effects using a basis-set approach, one needs to expand the wave function in terms of functions that explicitly depend on nucleus–nucleus, nucleus–electron, and electron–electron distances, i.e., the explicitly correlated functions (ECFs). In the first part of this work, we review the ECFs used in the atomic and molecular calculations of stationary bound states, and we discuss the features of these functions that may make them particularly useful in QM calculations of atomic and molecular systems. We particularly focus on Gaussian ECFs (ECGs), as these are the most popular functions used in non-BO atomic and molecular calculations.^6−20^ In the second part, two-dimensional time-propagation calculations are performed for two model systems involving Coulomb and Morse potentials using a grid approach. Next, the time-dependent grid wave functions are fitted with ECGs that are chosen to best represent the key features that appear in the wave function due to the interaction of the system with ultrashort intense laser pulses.

Single-particle Gaussians have been extensively used as basis functions in both electronic-structure theory^21^ and vibrational dynamics.^22−24^ In electronic-structure theory the Gaussians are real-valued functions centered at the atomic nuclei and contracted to form atomic orbitals which, in turn, form a nonorthogonal basis for the expansion of molecular orbitals—see, e.g., ref (21) for a detailed account. Such Gaussians have also been used for the study of many-electron dynamics,^25−27^ although important highly nonlinear phenomena such as ionization processes and high harmonic generation cannot be properly accounted for. The latter can to a certain extent be ameliorated by augmenting the standard basis with Gaussians fitted to continuum (Bessel, Coulomb, or Slater-type) functions; see the recent review by Coccia and Luppi and references therein for more details.^28^

The core idea of Heller’s approach^22−24^ to vibrational dynamics is to use complex-valued Gaussians (Gaussian wave packets), which are exact solutions for harmonic potentials. Unlike in electronic-structure theory, the Gaussian parameters are now time-dependent variational parameters. For anharmonic potentials, however, the equations of motion for the Gaussian parameters quickly become ill-conditioned and one resorts to locally harmonic approximations of the potential, which is a reasonable approach as long as the wave packet is sufficiently localized—see, e.g., ref (29) for a recent review of the so-called thawed Gaussian approach. Complex-valued Gaussians have also been used in the context of the multiconfigurational time-dependent Hartree (MCTDH) method.^30−33^ In all cases, however, the ill-conditioned equations of motion are a serious obstacle. In this work, we investigate the ability of complex-valued Gaussians to represent complicated dynamics of electrons and nuclei by fitting to accurate grid-based solutions of the time-dependent Schrödinger equation.

## Non-BO Hamiltonian

The total nonrelativistic all-particle non-BO molecular Hamiltonian describing the interaction of a neutral molecule with a uniform, time-dependent electric field defining the *x*-axis of a laboratory-fixed coordinate frame can be rigorously separated into a center-of-mass (COM) kinetic-energy operator and the internal Hamiltonian,^6,8,34^. The separation is accomplished by transforming the total Hamiltonian from Cartesian laboratory coordinates, **R**_*i*_, *i* = 1, ..., *N* (*N* is the total number of particles in the molecule) to a new Cartesian coordinate system, parallel to the laboratory frame, where the first three coordinates are the coordinates of the COM and the remaining coordinates are internal coordinates. The origin of the internal frame is chosen at a reference particle, typically the heaviest nucleus, which is taken to be particle number 1 such that ***r***_*i*_ = ***R***_*i*+1_ – ***R***_1_ for *i* = 1, ..., *n* with *n* = *N* – 1. The internal Hamiltonian then takes the following form (using atomic units throughout):^6^
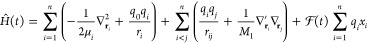
1where  is the time-dependent electric-field strength, *M*_1_ is the mass of the reference particle (particle 1), *q*_*i*_ = *Q*_*i*+1_ (*i* = 0, ..., *n*), μ_*i*_ = *M*_1_*M*_*i*+1_/(*M*_1_ + *M*_*i*+1_) (*i* = 1, ..., *n*) with *Q*_*i*_ and *M*_*i*_, *i* = 1, ..., *N*, the charge and mass of particle *i*, *r*_*ij*_ = |**r**_*i*_ – **r**_*j*_| = |**R**_*i*+1_ – **R**_*j*+1_|, *r*_*i*_ = |**r**_*i*_|, and the prime denotes vector/matrix transposition. The Hamiltonian (1) represents *n* interacting particles with masses equal to the reduced masses moving in the central Coulomb potential of the reference particle. We refer to these particles as pseudoparticles because, while they have the same charges as the original particles, their masses are different. For  the Hamiltonian (1) is fully symmetric (isotropic or atom-like) with respect to all rotations around the center of the internal coordinate system and its eigenfunctions transform as irreducible representations of the fully symmetric group of rotations. When , however, the symmetry is reduced to cylindrical about the field direction, here the *x*-axis.

For a diatomic system, after separation of the center of mass motion, the internal Hamiltonian used in the non-BO time-evolution calculations represents a motion of the second nucleus and the electrons (with their masses replaced by the respective reduced masses) in the field of charge of the first nucleus (the reference nucleus) located in the center of the internal coordinate system. The potential acting on the second nucleus that results from the interaction of this nucleus with the charge of the reference nucleus and the electrons can effectively be represented by a Morse-like potential. An important effect that also determines the electronic-nuclear dynamics of the system is the electrostatic attractive interaction of each of the electrons with the reference nucleus located at the center of the coordinate system. Thus, the Coulombic and Morse interactions investigated in this work are central to understanding the molecular dynamics. The interactions, which are present in the internal Hamiltonian but are not investigated in this work, are two particle interactions involving the second nucleus and the electrons and the interelectron interactions. To represent these types of interactions, models involving more than two dimensions would be needed, and thus, they are not investigated in this work. However, based on our ECG stationary-state calculations, we expect that FFECGs should perform very well in describing these interactions.

## ECGs Used in Very Accurate Non-BO Calculations of Stationary States of Small Atoms and Molecules

To achieve the high accuracy needed in the computations, we will use an approach that is both adaptive in space and time. Mesh-free complex explicitly correlated Gaussian (CECG) functions, which are free to warp and roam in space, will be the main tool. The Adamowicz group has used various types of ECGs and CECGs in very accurate non-BO atomic and molecular calculations of stationary bound states for more than two decades. Various forms of explicitly correlated all-particle Gaussian functions (ECGs) with real and/or complex nonlinear parameters have been used in non-BO calculations.^6−9^ The simplest ECG with real nonlinear parameters used to calculate an *S* state of an *n*-electron atom (*S*-ECG) is
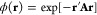
2where **r** is vector of 3*n* internal Cartesian coordinates of the electrons and **A** is a 3*n* × 3*n* real symmetric positive-definite matrix of the nonlinear parameters. **A** has the following block structure: **A** = **a** ⊗ **I**_3_, where **a** is a *n* × *n* real dense symmetric positive-definite matrix and **I**_3_ is a 3 × 3 identity matrix, while symbol ⊗ denotes the Kronecker product. Such a representation of matrix **A** ensures that the exponential part of the basis function is invariant with respect to 3D rotations.

*S*-ECGs can alternatively be represented as
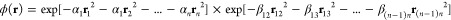
3where the first factor is a product of *n* orbitals and the second factor is a product of *n*(*n* + 1)/2 pair functions explicitly dependent on the squares of all interelectron distances, . The methods allow for very accurate calculations of the spectra of small atoms and molecules when the leading relativistic and QED corrections are also included in the calculations.

The non-BO ECG calculations for *S*, *P*, *D*, and *F* states of atomic systems with 2–5 electrons^35−47^ are among the most accurate in the literature. As the ECGs explicitly depend on the distances between the particles (electrons and nuclei), they very efficiently represent the coupled nucleus-electron motions and allow very accurate accounting of the interparticle correlation effects. These effects are indispensable in non-BO calculations because, as mentioned, the Coulomb interactions make particles with alike charges avoid each other and particles with opposite charges follow each other. This effect can also be very effectively described with the ECGs.

A challenge in non-BO ECG calculations of stationary ground and excited states of small atoms and molecules is to accurately describe radial and angular oscillations of the non-BO wave functions of highly excited states. Three types of ECGs were used to describe these features. These are as follows:

a molecular ECGs with pre-exponential multipliers in the form of powers of the internuclear distances. The functions are called “power” ECGs (PECGs) and they have the following form:
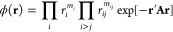
4where *m*_*i*_ and *m*_*ij*_ are even non-negative integers, and **A**, as defined before, is a real symmetric positive-definite 3*n* × 3*n* matrix. PECGs have been used in molecular non-BO calculations for small molecules;^44,48−53^
b complex single-center ECGs. The works that are particularly relevant to this project concern implementation of algorithms for performing very accurate calculations on bound states of small molecules that employ complex ECGs (CECGs):
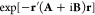
5where **A**, as defined before, is a real symmetric matrix and **B** is also a real symmetric matrix with the same structure as **A** (i.e., **B** = **b** ⊗ **I**_3_, where **b** is a real dense symmetric *n* × *n* matrix). It was shown that CECGs can very effectively describe high-frequency radial oscillations of the wave function of highly vibrationally excited states. The angular oscillations can be described by adding Cartesian spherical harmonics as pre-exponential multipliers to the Gaussians. CECGs have been used in non-BO calculations of molecular Σ, Π, and Δ bound rovibrational states^54−57^ It has been shown that CECGs are equally, if not more, efficient as PECGs in describing radially and angularly oscillating wave functions of states located near the dissociation threshold;
c real ECGs with shifted centers (SECGs) of the form:
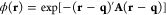
6where **q** is 3*n* real vector of the Gaussian shifts and **A**, as defined before, is a real symmetric matrix of the nonlinear parameters. SECGs have been used in non-BO calculations of some small diatomic and triatomic molecules and in non-BO calculations of the dipole moments, polarizabilities, and hyperpolarizabilities of isotopologues of the H_2_ molecule.^58−65^ Including real shifts in the Gaussians enables one to describe radial and angular polarization of the wave function. These types of deformations can also be described by linear combinations of spherical-harmonics factors, though the shifts may be a more effective way for the task.

The purpose of this work is to develop and test an ECG basis set to be employed in quantum-dynamics simulations of molecular systems exposed to an ultrashort laser pulse within the semiclassical electric-dipole approximation. The proposed basis is tested by fitting wave packets obtained as solutions of the time-dependent Schrödinger equation with a grid-based method for two two-dimensional (2D) model systems. The models represent two main features that need to be described in a QD simulation of a molecule, i.e., the electrostatic interaction represented by a Coulombic potential and the rovibrational interaction represented for a diatomic molecule by a Morse potential. In the next section, before the ECG basis functions for QD molecular simulations are introduced, grid-based calculations of the two models are described and discussed.

## 2D Model Calculations Using a Grid Approach

Our ultimate goal is to solve the TDSE, , for the non-BO internal Hamiltonian (1) representing a molecule interacting with a short intense laser pulse. For a diatomic molecule, there are two major interactions that must be described. The first is the repulsive interaction of the pseudonucleus with the charge of the reference nucleus located in the center of the coordinate system, and the second is the attractive Coulomb interaction of a pseudoelectron with the charge of the reference nucleus. Due to the screening effect of the former interaction by the electrons, the interaction potential is not Coulombic but is more appropriately represented by a Morse-type potential. Thus, at the very minimum, in selecting an ECG basis set for solving the non-BO TDSE, one should verify whether the chosen basis is capable of describing the laser-induced dynamics of a single particle interacting with the charge of the reference particle with the Morse and Coulomb potentials. For the verification, we use an elementary 2D model Hamiltonian of the form:
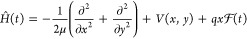
7

**Figure 1:**
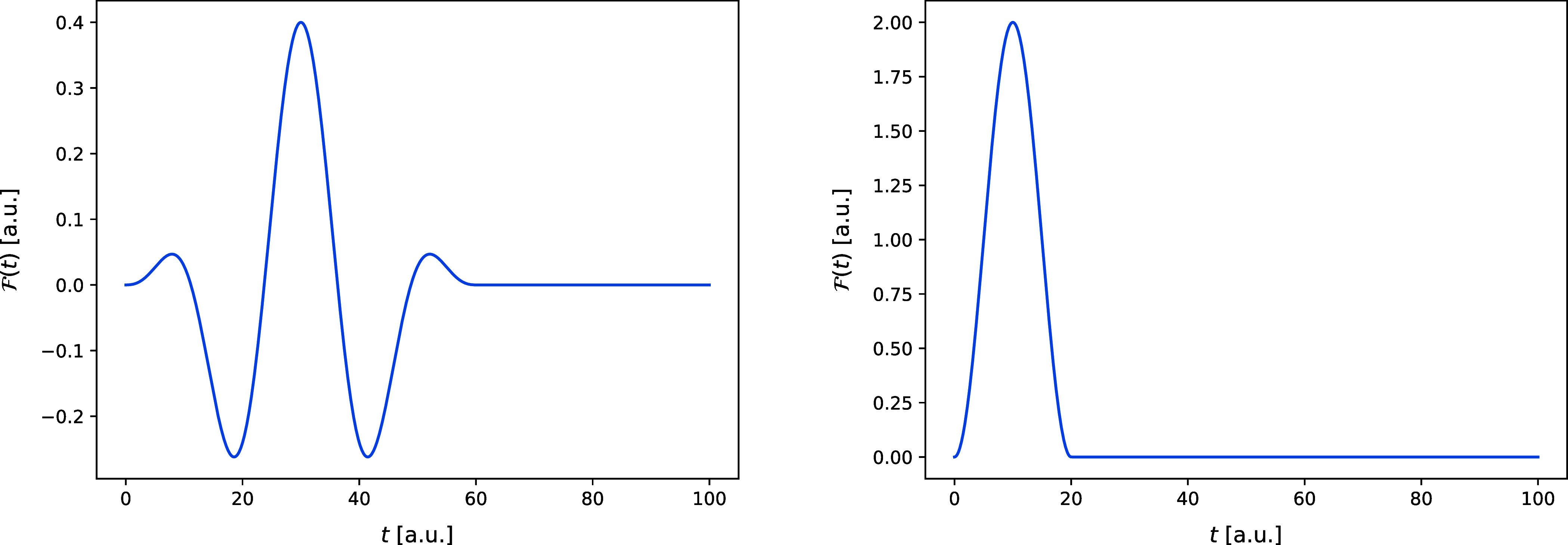
Shape of the laser pulse used in the simulation of the Coulomb model (left) and of the Morse model (right).

For the electron we use the soft Coulomb potential, , which mimics the nuclear potential of a hydrogen-like atom, and the Morse potential is given by , with *D*_*e*_ = 0.17449, *r*_*e*_ = 1.4011, and α = 1.4556. The charge and (reduced) mass are set to *q* = −1, μ = 1 for the Coulomb model, and *q* = 1, μ = 1605.587 for the Morse model. The electric-field strength is nonzero only in the time interval *t*_0_ < *t* < *t*_1_, where it is equal to
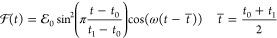
8where  denotes the maximum electric field amplitude. In our calculations for both models, we set *t*_0_ = 0. For the Coulomb model we set ω = 0.25 au, *t*_1_ = 60 au, and , which corresponds to a laser pulse of wavelength  nm, consisting of 2.5 optical cycle. For the Morse model we set ω = 0.0 au, *t*_1_ = 20 au and , corresponding to a short, delta-like pulse (relative to the time-scale of the nuclear motion). Our laser pulse parameters are chosen not for their significance in relation to any experiment but rather such that it generates complicated ionization and dissociation dynamics. We consider the dynamics for times 0 ≤ *t* ≤ 100 au for the Coulomb model and 0 ≤ *t* ≤ 300 au for the Morse model, including periods of free evolution after the laser pulse. The laser pulses used in the QD simulations of the Coulomb and Morse models are shown in Figure 1.

**Figure 2:**
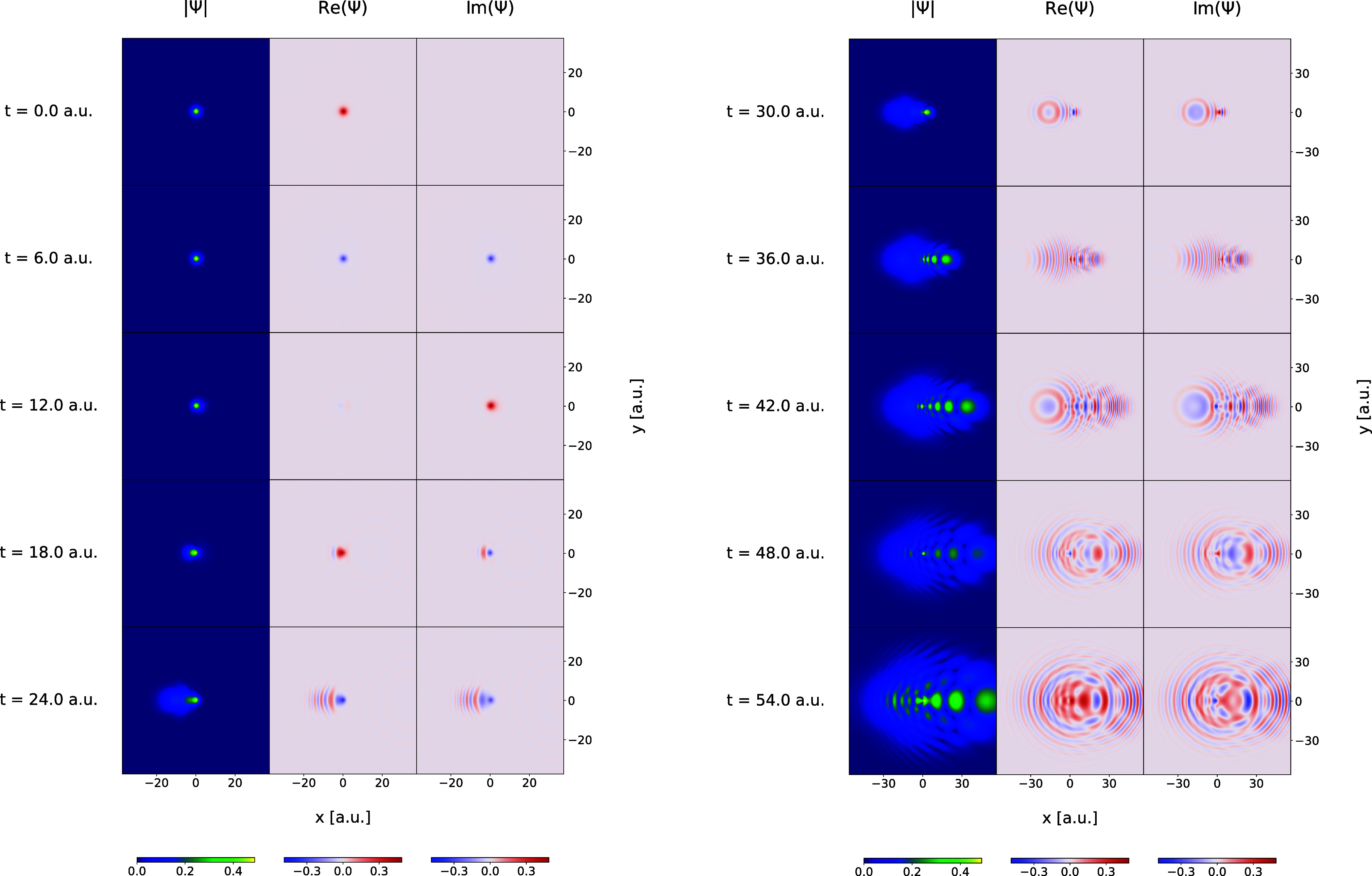
Snapshots illustrating the time evolution of the Coulomb model wave function during the grid-based simulation in the time interval from *t* = 0.0 to *t* = 54.0 au As the time advances, the wave function becomes progressively more complicated, with nonlinear phase, amplitude oscillations, and localized features. To facilitate visualization, the wave function values have been rescaled so that the maximum value remains constant across all timeframes, matching the maximum value at *t* = 0.

**Figure 3:**
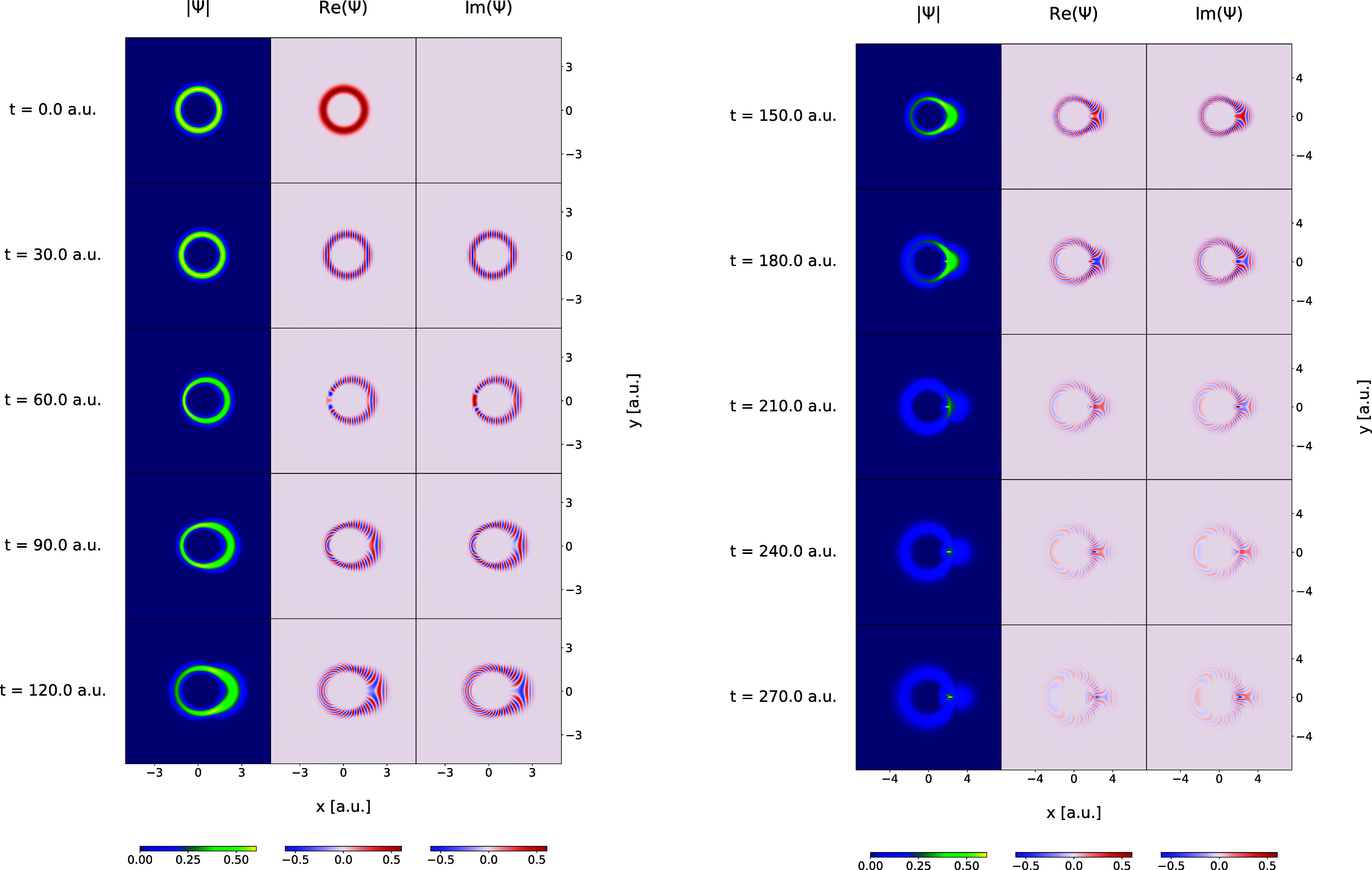
Same as in Figure 2 but for the Morse model in the time interval from *t* = 0.0 au to *t* = 270.0 au.

Highly accurate reference QD calculations with the Coulomb and Morse potentials are performed by spatially discretizing the real *xy* plane using a grid with *n*_grid_ = 1024 equidistant points in the interval [−*L*, *L*] = [−150, 150] for the Coulomb model and [−20, 20] for the Morse model, in both directions. The kinetic-energy operator is approximated using the standard Fast Fourier Transform (FFT), which introduces artificial periodic boundary conditions. These have a negligible effect due to the large domain. The time evolution can be approximated in a number of ways, but we choose the common second-order split-operator scheme^66^ with time step *h* = 0.01 au for the Coulomb model and *h* = 0.05 au for the Morse model, respectively. This method has accuracy of order  locally in time, and is sufficiently accurate for our purposes. The calculations are initiated with the corresponding ground-state wave functions, which are fully symmetric with respect to all rotations around the center of the internal coordinate system in the *xy* plane. The ground-state wave function is obtained using inverse iterations using the conjugate-gradient method for the solution of large sparse linear systems. In the case of the Coulomb potential the ground-state wave function approximates a 2D 1*s* orbital and, in the case of the Morse potential, the ground-state wave function has a “torus” shape and is practically zero at the coordinate center, peaks at *r*_*e*_, and then again goes to zero at larger distances. In Figures 2 and 3, some snapshots of the time evolution of the wave functions are shown for the Coulomb and Morse simulations, respectively. For each case, the real and imaginary parts of the wave function, as well as the wave function absolute value, are plotted. In both cases, the respective wave functions become increasingly more complicated with many features, more deformed and oscillatory, and more diffused.

It is interesting to know to what extent the time-evolving wave packets for the two considered models involve contributions from higher angular momenta. For both systems, at *t* = 0, the initial wave packets, i.e. the corresponding wave functions, are fully rotationally symmetric, i.e., for both , where  is the angular momentum operator in 2d and θ is the angular coordinate in planar polar coordinate system. Moreover,  before and after the pulse begins and after it ends. The nonvanishing of the commutator for *t*_0_ < *t* < *t*_1_ implies that ψ(*t*) is not, in general, an eigenfunction of .

**Figure 4:**
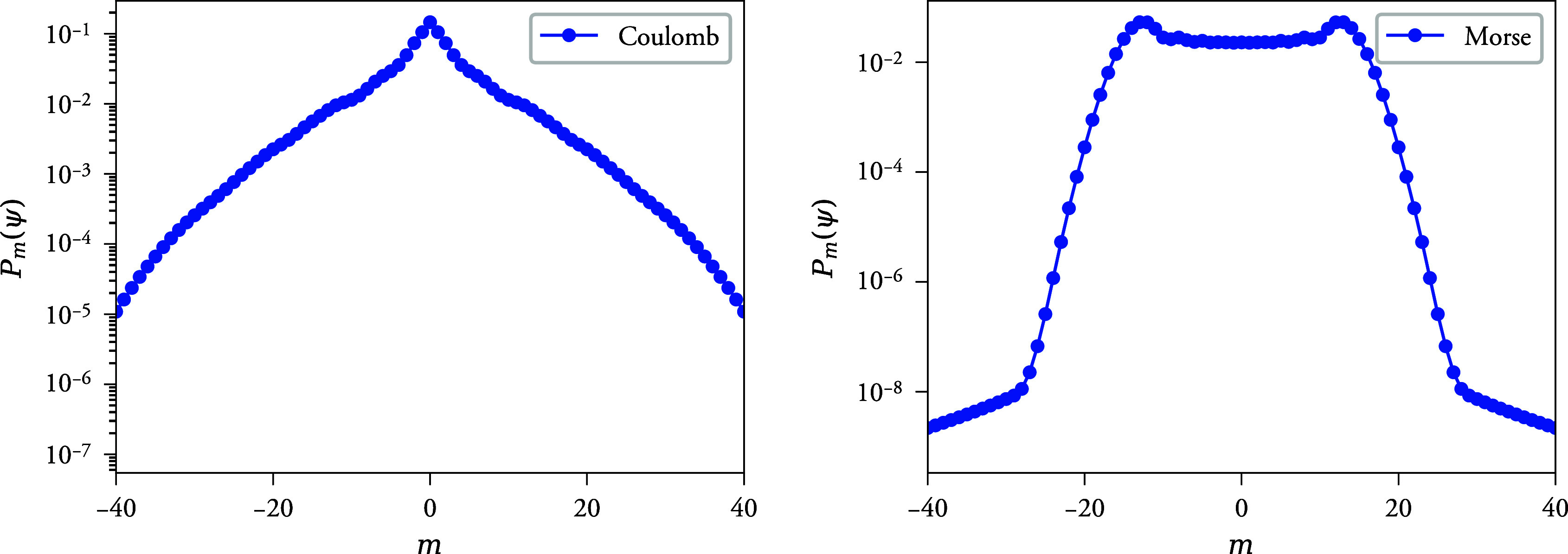
Left: Angular momentum probability distribution of the Coulomb model wave function at *t* = 45. Right: Same for the Morse model at *t* = 20 au While the Morse model is bimodal with strong peaks around |*m*| = 13, the Coulomb model is unimodal, with standard deviation σ_*m*_ = 6.9. The spectra are computed by first sampling the grid wave function at a polar coordinate grid using high-order spline interpolation, and then Fourier decomposing the result along the angular coordinate, resulting in ψ(*t*) = *∑*_*m*_(2π)^−1/2^*e*^*imθ*^*f*_*m*_(*r*, *t*). The probabilities plotted are defined as , and are computed using numerical quadrature.

In Figure 4, the angular momentum probability distributions are shown for both model systems. Clearly, the pulse induces high angular moments in the wave function.

Fitting of the wave functions obtained in the Coulomb and Morse simulations with ECGs is described in the next section. Based on the analysis of the simulation results, it is clear that Gaussians used in the fitting need to be capable of describing the ground-state wave function, the cylindrical deformation and oscillation of the function due to the interaction with the field, the diffusion of the function associated with possible ionization or/and dissociation of the system, and the coupling of the motion of all particles forming the system including the non-BO coupling of the motions of the electrons and the nuclei. Most of these features appear in the calculations of molecular static ground and excited states, and an excellent performance of the ECGs has been well documented in those calculations. The features that do not appear in the static calculations are ionization and dissociation. It necessitates that the wave function is allowed to acquire some plane-wave character. This can be achieved by allowing the shift vector and parameter matrix **A** in Gaussian (5) to become complex. Such Gaussians, named by us the fully flexible explicitly correlated Gaussians (FFECGs), have the following form:
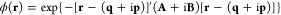
9or alternatively the following form:
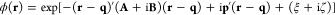
10where **A** and **B**, as defined before, are real symmetric 3*n* × 3*n* matrices, and **p** and **q** are 3*n* vectors. The FFECGs are fully flexible complex multiparticle Gaussians that can provide a basis set for expanding a time-evolving non-BO wave packet of a molecular system interacting with a short fast-varying intense laser pulse. As shown in the next section, a linear combination of FFECGs can be used to very accurately represent the ground-state wave functions of the two models considered in this work. It can also describe the time-dependent oscillations of the time-evolving wave packet. Also, due to the fact that the Gaussian shift vectors are complex, the ionization and dissociation processes can be described. And finally, FFECGs can very effectively represent the coupled and highly correlated motions of the electrons and nuclei forming the system. They allow the electrons and the nuclei to be treated on equal footing in the calculation.

## Fitting the Grid-Based Wave Packet with FFECGs

The 2D wave packet obtained in the time-dependent grid calculation at each particular time step for each of the two models is fitted with a linear combination of FFECGs. In this case, an FFECG has the following form:
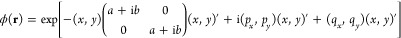
where **p** = (*p*_*x*_, *p*_*y*_)′ and **q** = (*q*_*x*_, *q*_*y*_)′. Thus, in the 2D case ϕ(**r**), is dependent on six real nonlinear parameters, *a*, *b*, *p*_*x*_, *p*_*y*_, *q*_*x*_, and *q*_*y*_. As the time-dependent calculations for both models are initiated with the corresponding ground-state wave functions, which are real and spherically symmetric, appropriate FFECGs need to be used. For the hydrogen model, these FFECGs are simple spherical Gaussians centered at *q_x_* = *q_y_* = 0 with *a* ≠ 0 and *b* = *p*_*x*_ = *p*_*y*_ = 0. A linear combination of FFECGs of this kind can fit the ground-state wave function obtained in the grid calculation with very good accuracy. For the Morse model, one needs to use only FFECGs with *a* ≠ 0, *b* ≠ 0, and *p*_*x*_ = *p*_*y*_ = *q*_*x*_ = *q*_*y*_ = 0 to generate a good fit to the ground-state wave function obtained in the grid calculation. Additionally, for each FFECG pair used, the two Gaussians need to have the same *a*, but their *b*’s should be *b* and −*b*, so they can be “contracted” to form the following real function:
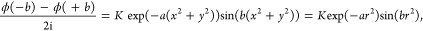
11where *K* is a constant. A linear combination of such contracted FFECGs provides a very accurate fit to the ground-state wave function for the Morse model.

**Figure 5:**
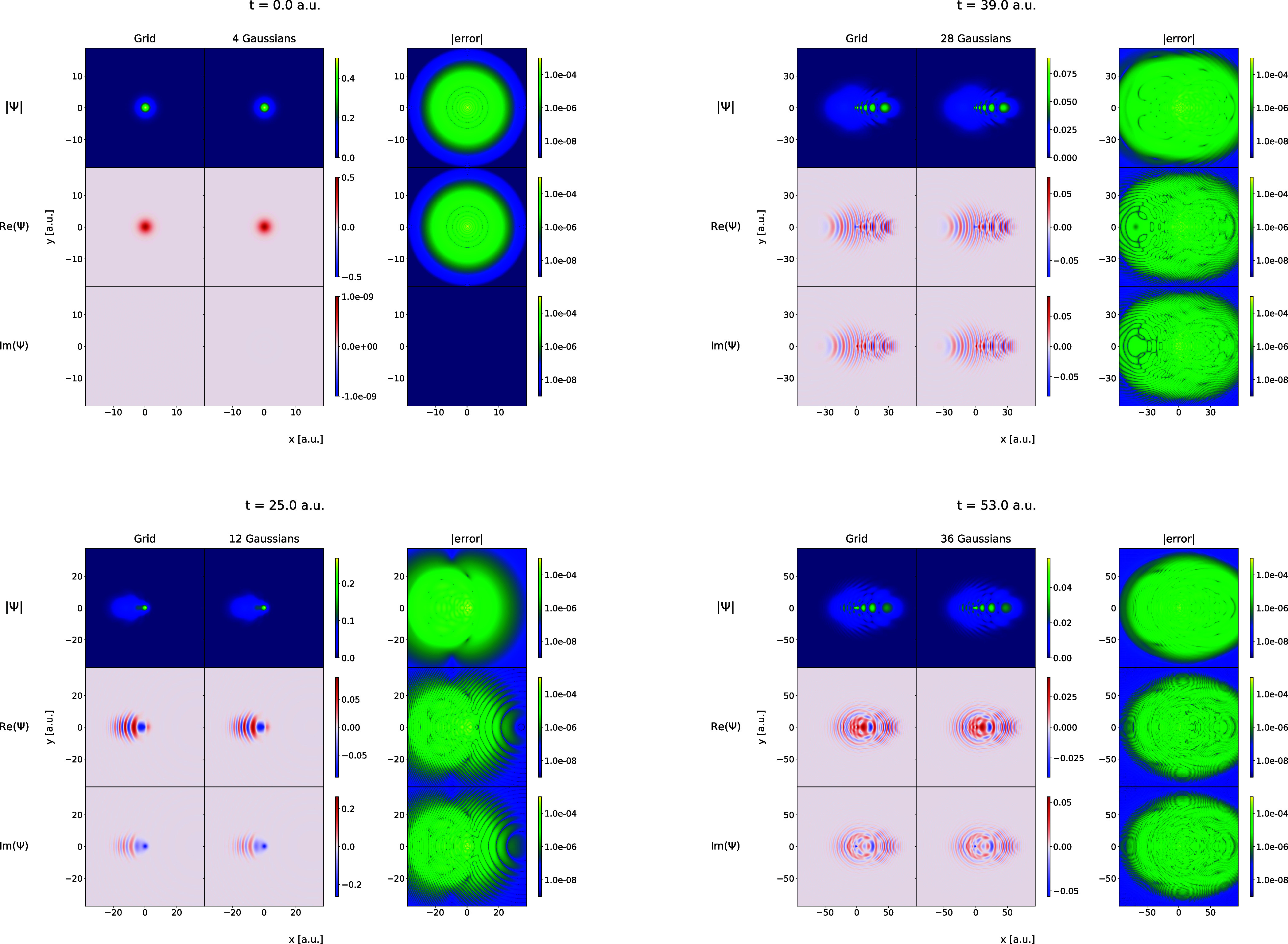
FFECG fits to the selected time frames extracted from the grid-based simulation trajectory of the Coulomb model. The error represents the difference in either the absolute value, the real part, or the imaginary part between the grid wave function and the optimized linear combination of Gaussian functions.

**Figure 6:**
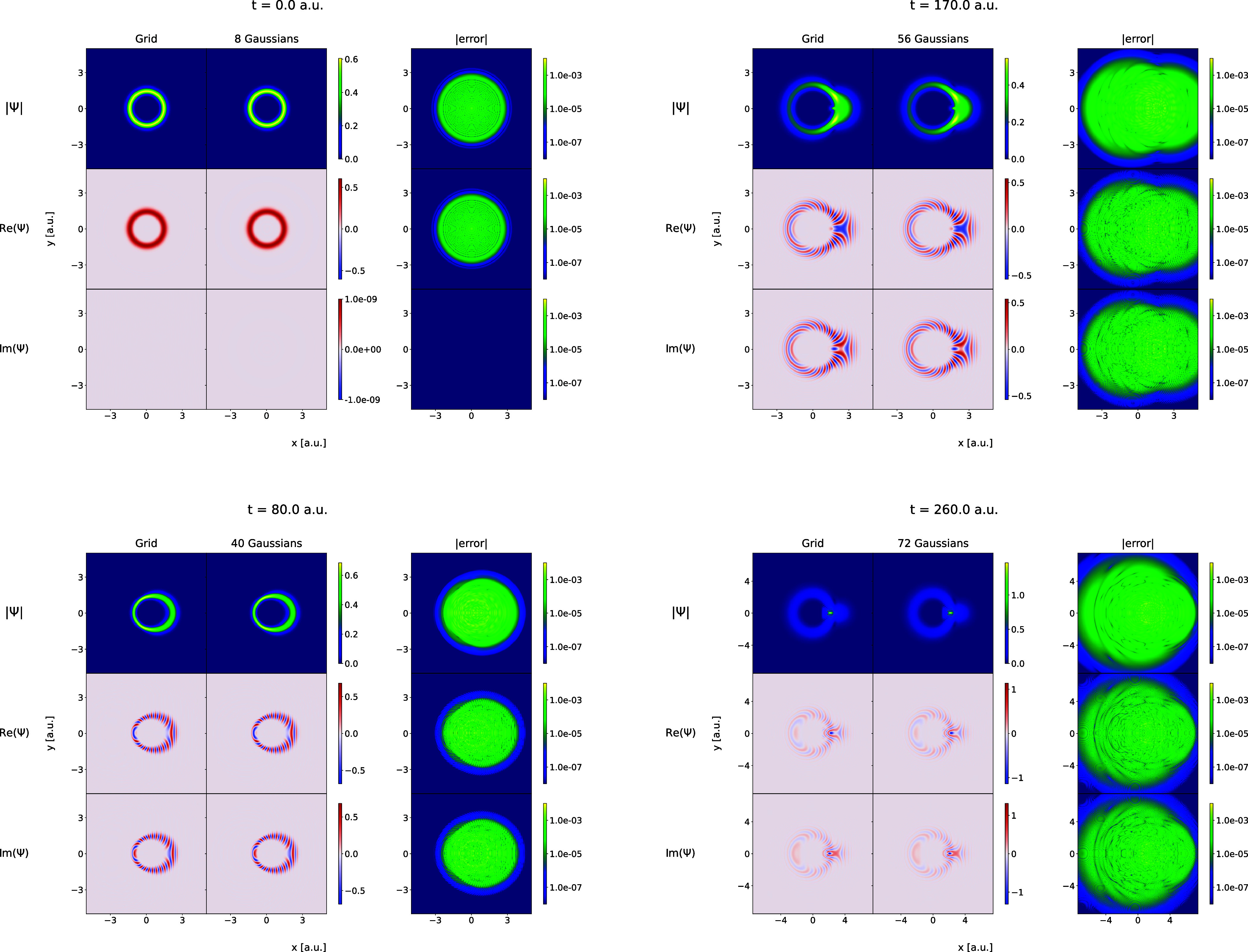
Same as Figure 5 but for the Morse model.

The fitting of the grid ground-state wave functions with FFECGs is done using the standard least-squares implementation from the SciPy Python library.^67^ The method is also used to fit the wave functions obtained in consecutive time steps in the grid simulation. First, the number of FFECGs used is the same as that for the ground state, but the parameters frozen at zero for the ground-state wave function are now unfrozen and optimized. For both models we set the threshold for the least-squares cost function equal to 10^–5^. When the assumed accuracy of the fitting cannot be achieved, additional FFECGs are added to the basis set. Each addition includes a group of FEECGs with the following parameters:
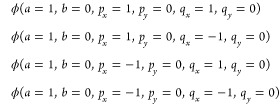


The fitting that involves optimization of all linear and nonlinear parameters of the enlarged FFECG basis set continues for some number of the following time steps until it is determined that the fitting process is no longer successful. At that point, new FFECGs are added to the basis set, and the wave packet is refitted. The fitting for the Coulomb and Morse models is shown in Figures 5 and 6. In the figures, the wave packets obtained in the grid calculations are compared with the corresponding FFECG fits for some selected time points.

**Figure 7:**
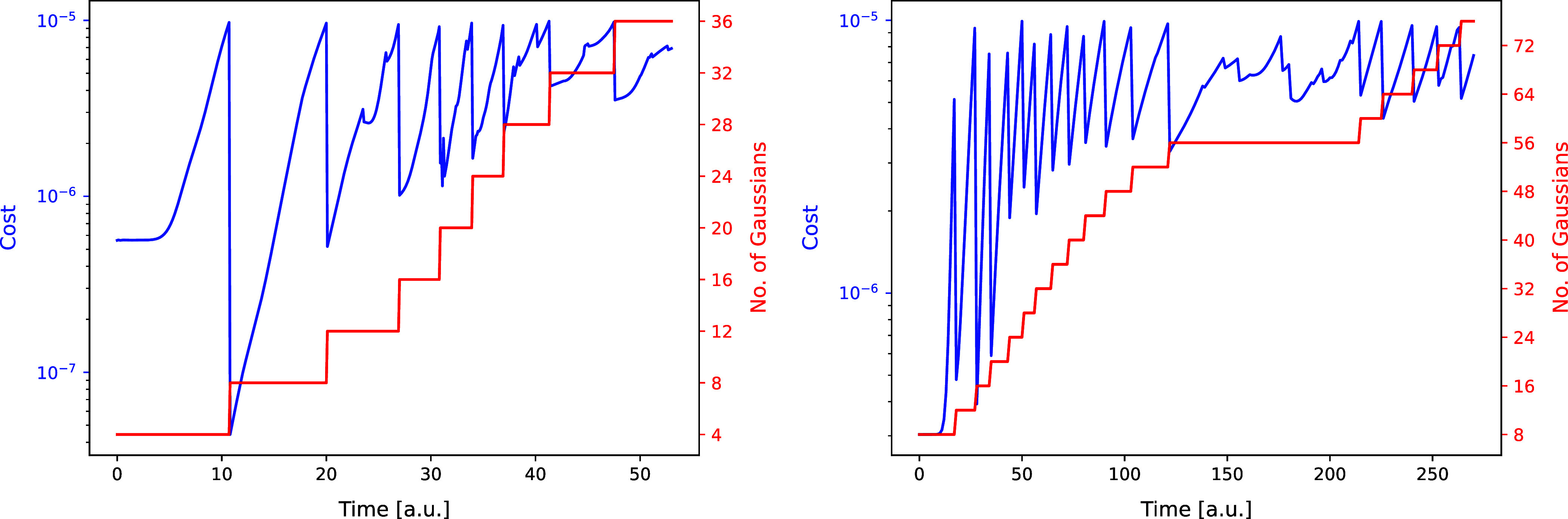
Evolution of the cost function and FFECG basis set size over time during the fitting process for the Coulomb model (left) and for the Morse model (right). A new set of functions is added to the total basis set whenever the cost function reaches the predetermined threshold of 10^–5^, ensuring the maintenance of the desired level of accuracy.

Also, in Figure 7 the changes of the cost function representing the accuracy of the fitting, as well as the time-resolved size of the basis set, are plotted for the Coulomb and Morse models. Figures 5–7 show that the fitting accuracy is satisfactory for both models despite the wave function becoming increasingly more complicated. Naturally, as this happens, the number of FFECGs has to be constantly increased.

In general, the Morse model seems to be somewhat more difficult to represent using FFECGs than the Coulomb model. The reason for this behavior can be attributed to the maximum of the density of the second nucleus in the Morse model being shifted away from the reference nucleus by some distance (in the ground state, this distance is approximately equal to the equilibrium internuclear distance). This type of shifting does not happen in the Coulomb model. The shifting of the density maximum away from the reference nucleus required the inclusion of more ECGs in the wave function. However, if this is done, the ECG expansion of the wave packet in the Morse model should be equally accurate as it is for the Coulomb model.

The least-squares fitting of a linear combination of ECGs to a grid wave function produces a wave packet that provides a representation of the grid function with uniform spatial quality. ECG representations obtained by minimization of energy-based functionals (e.g., the variational Rayleigh–Ritz functional or Rothe variational functional) usually are more accurate in some spatial domains than the others. It is difficult to *a priori* determine which imperfections of the representation of the wave function will be amplified and which will be suppressed when a particular observable is calculated.

**Figure 8:**
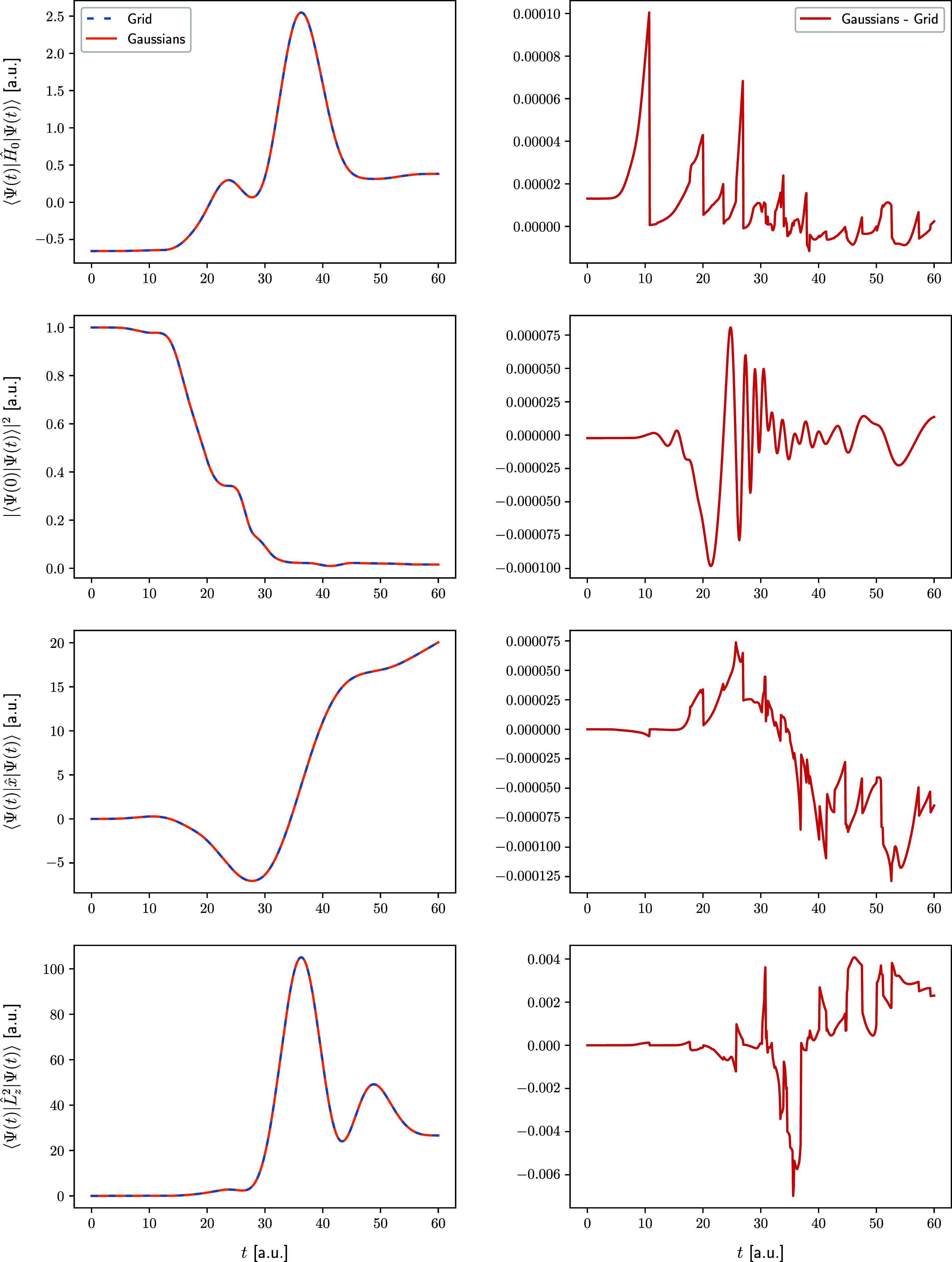
Left: Time-resolved observables computed for the Coulomb model, with the reference grid wave function (dashed lines), and with the fitted Gaussian wave function (solid lines). Right: Differences between grid and Gaussian observables. From top to bottom: expectation value of the field-free Hamiltonian, projection of the wave packet onto the ground-state wave function (the initial state), dipole moment expectation value, expectation value of the squared *z*-component of the angular momentum.

**Figure 9:**
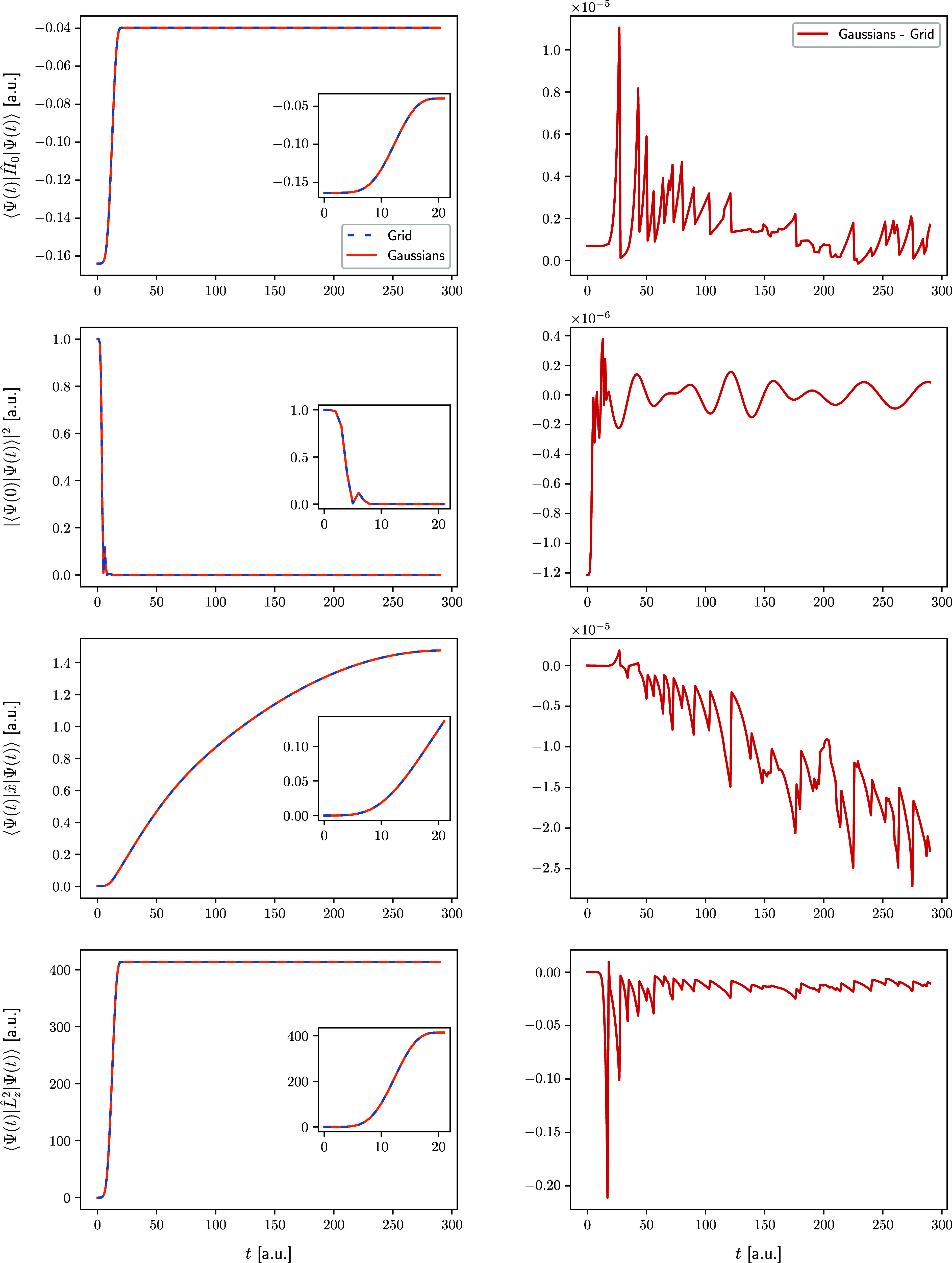
Same as Figure 8 but for the Morse model. The insets display the evolution of the observables over the duration of the laser pulse (0–20 a.u).

To better elucidate the accuracy of the FFECG fits obtained in this work, we compare time-resolved observables obtained for the grid wave packet and the fitted wave packet. The calculated observables are the expectation value of the field-free Hamiltonian, the ground-state survival probability (i.e., the square of the autocorrelation function), the dipole moment expectation value, and the expectation value of the squared *z*-component of the angular momentum. The comparison is shown in Figure 8 for the Coulomb model and in Figure 9 for the Morse model. On the left-hand side of each figure, the time-resolved plots of each of the four observables for the grid and FFECG wave packets are shown together in four separate frames. As one can see, within the left-hand-side plots, the curves corresponding to the two wave packets for all four observables for both models are practically indistinguishable. The right-hand-side plots do indicate some error fluctuations, but these are of similar magnitude as the fluctuations in the fitting error and, therefore, are acceptable.

We note that the ground-state survival probability of the final state is small or zero for both models and that the angular-momentum expectation value indicates involvement of highly excited rotational states. The final energy for the Coulomb model is positive, indicating an unbound (i.e., ionized) state. For both models, the increase of the dipole moment during the dynamics indicates the large, asymmetric spreading of the wave packet. It is quite remarkable that so few FFECGs are required to accurately reproduce such complicated dynamics.

## Conclusion

The grid approach is used to obtain solution of the time-dependent Schrödinger equation for two 2D model systems that represent features which appear in quantum-dynamics time-propagation of the wave packet representing a diatomic neutral molecule interacting with a short intense laser pulse and performed without assuming the Born–Oppenheimer approximation. The grid wave functions obtained in consecutive time steps are fitted with a combination of Gaussian functions that are 2D versions of the more general fully flexible explicitly correlated Gaussians (FFECGs) with complex exponential parameters and complex shifts of the Gaussian centers. The fitting procedure employs the least-squares method and involves growing the basis set of the Gaussians to provide a uniformly good fit for a representative set of time points obtained from the grid time propagation. The two models considered in the calculations involve a single-particle in the central potential represented by an attractive Coulomb interaction and a Morse potential. Based on the results obtained in the calculations, we can expect that FFECGs will provide a good basis set for laser-induced non-BO dynamics of a diatomic molecule. Work on implementing FFECGs in molecular QD simulations is in progress.

Finally, this work represents a preliminary step in the application of FFECGs to describe the coupled electronic-nuclear dynamics in atomic and molecular systems. In the future work involving FFECGs and the non-BO nuclear-electronic quantum dynamics, the Rothe method^68−70^ will be employed to propagate the wave packet. The approach, also known as the adaptive method of time layers,^71^ relies on reformulating the time-dependent variational principle into a series of minimizations of the Rothe functional at consecutive time steps. This is an alternative to the standard real-time propagation techniques based on the Dirac–Frenkel variational principle and propagated using, e.g., Runge–Kutta methods. The ECG optimization protocol developed in the present work to fit a linear combination of ECGs to the grid-based wave packet will be applied to minimize the Rothe functional with respect to the linear and nonlinear parameters of the ECGs. In our works on the variational calculations of molecular stationary states with real and complex ECGs we have developed procedures for the variational minimization of the energy functional, which employs the analytical energy gradient determined with respect to the ECG nonlinear parameters. The use of the gradient has significantly expedited the functional minimization and enabled non-BO energies and the corresponding wave functions whose accuracy by far exceeds the results obtained by others. The gradient-based approach will also be used in the optimization of the ECG parameters carried out through the minimization of the Rothe functional. The high efficiency of the computer code for the optimization of the ECGs in the stationary-state calculations has been also achieved by deriving the algorithms for calculating the necessary N-particle matrix elements (i.e., the overlap, Hamiltonian, and gradient matrix elements) using the matrix differential calculus and by coding them using highly parallel, vectorized, and GPU-enabled strategies. These strategies will be used in the Rothe time-propagation calculations.
